# From Standard Establishment to Precision Intelligence: Research Progress in Quality Control of Mammography

**DOI:** 10.3390/diagnostics16050651

**Published:** 2026-02-24

**Authors:** Hongyang Du, Yuxi Zhou, Mingming Ma, Yuan Jiang, Naishan Qin

**Affiliations:** Department of Radiology, Peking University First Hospital, Beijing 100034, China

**Keywords:** mammography, quality control, technological evolution, standardization, artificial intelligence

## Abstract

Breast cancer remains the most common female cancer globally, and mammography is pivotal for its early screening and diagnosis, with quality control (QC) as a core guarantee. Mammography QC has advanced from early technical exploration to whole-process management, driven by technological innovation and standardized protocols. This review summarizes the historical evolution, influencing factors, corresponding measures, limitations, and prospects of mammography QC to promote the construction of standardized management systems.

## 1. Introduction

Since the inaugural global cancer burden assessment was published in the 1980s, breast cancer has consistently ranked as the most frequently diagnosed malignancy among women worldwide, posing a grave threat to female health and imposing a substantial social burden. Over the past three decades, the global incidence rate of breast cancer has increased by 57.8%, with an average annual growth rate of approximately 0.5% [1]. In 2022 alone, there were 2.3 million newly diagnosed cases and 670,000 deaths worldwide [2]. Common imaging examination methods for the breast include mammography, ultrasound, and magnetic resonance imaging (MRI). Mammography uses differences in X-ray absorption among tissues to detect abnormal findings in the breast, such as masses, calcifications, and structural distortions. It is particularly sensitive to microcalcifications and is currently the most evidence-based imaging technology for reducing breast cancer mortality [3]. The American College of Radiology (ACR) recommends annual mammography screening for women starting at age 40 [4], but because the examination involves ionizing radiation, ensuring optimal image quality to reduce radiation exposure is especially crucial. For dense breasts, ultrasound serves as a valuable adjunct to mammography, as it is independent of breast density. However, ultrasound is limited in detecting deep-seated lesions and microcalcifications, and its diagnostic accuracy is highly operator-dependent. In high-risk populations, such as BRCA1/2 mutation carriers, MRI is added to mammography and ultrasound protocols to improve diagnostic sensitivity [5]. Nevertheless, due to high costs, long acquisition times, and a high false-positive rate, MRI is not suitable for routine screening of the general population.

Multimodal imaging optimizes early breast cancer detection by leveraging the strengths of each technique, with mammography remaining a cornerstone of clinical practice. Image quality is the foundation for accurate radiologic diagnosis, defined by technical parameters such as signal-to-noise ratio (SNR), spatial resolution, and contrast [6]. Quality control (QC) in mammography is the core guarantee of high-quality images and is susceptible to multiple factors, including equipment, technologists, physicians, patients, and the environment. Focusing on issues such as inconsistent global protocols, subjective variability, and limited adaptability to emerging technologies in mammography QC, this review systematically traces the historical evolution and key determinants of QC systems, further analyzes current limitations, and envisions the future of precision intelligence, offering insights for the construction of a standardized QC management framework.

## 2. Historical Development of Mammography QC

From the early use of traditional tungsten-target X-ray tubes for mammography to the emergence of dedicated mammography machines and the subsequent adoption of full-field digital mammography (FFDM) systems, the development of mammography QC has been closely linked to advances in breast imaging technology and the renewal of quality management concepts, evolving from simple initial technical operations to whole-process management today.

### 2.1. Early Exploration and Technical Foundation of Mammography QC

**Figure 1:**
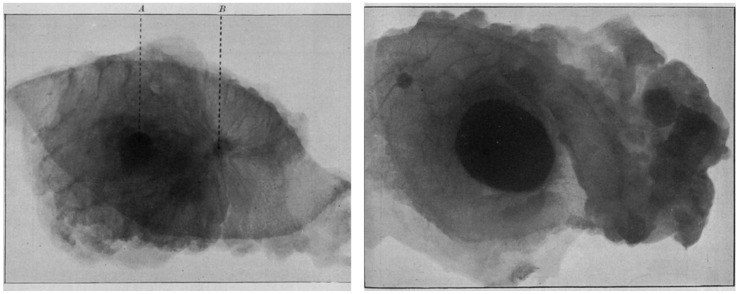
Mammographic images of breast tissue specimens by Salomon Albert in 1913. (**Left**) Chronic mastitis complicated with solid carcinoma. A. Nipple. B. Solid carcinoma with radiating extensions. (**Right**) A well-circumscribed cancer mass is visualized in the mid-field. Malignant lymphadenopathy is identified within the right axillary adipose tissue. Reprinted with permission from: [7].

The history of mammography can be traced back to 1913, when Salomon first performed X-ray imaging on breast tissue specimens and confirmed the X-ray characteristics of breast cancer (Figure 1) [7]. In the 1950s, Leborgne identified the diagnostic value of microcalcifications for breast tumors [8] and proposed that image quality could be improved by breast compression and immobilization [9]. However, limited by the performance of equipment at that time, mammography QC was still in the stage of early exploration and technical foundation-laying. The advent of Egan’s mammography technique [10] and the first dedicated mammography machine, Senographe, in 1965 [11] promoted the clinical application of mammography. In 1971, Shapiro et al. reported findings from a randomized controlled trial in which they compared regular mammography screening and clinical breast examination against routine medical care alone. The study revealed a significant reduction in breast cancer mortality in the screened group at 3.5 years, providing the first robust evidence that mammography screening reduces breast cancer mortality [12]. Concurrently, there was a growing recognition that image quality exerts a significant impact on diagnostic accuracy [13].

### 2.2. Establishment of a Standardized Mammography QC System

In the 1980s, the ACR launched the first mammography accreditation program, which put forward basic requirements for equipment, radiation dose, and personnel qualification, representing the initial standardized attempt at mammography QC [14]. However, being voluntary in nature, it had relatively few participating institutions in the early stages. In 1992, the U.S. Congress enacted the Mammography Quality Standards Act (MQSA), making the ACR’s accreditation program a mandatory requirement. In addition to the basic requirements, it also required the introduction of final assessment grading and recommendations, patient result notification, follow-up review, and annual inspections of accredited institutions [15]. During this period, significant advancements were made in improving image quality and optimizing radiation dose in mammography [16], which also promoted the reclassification of breast lesions. In 1992, the ACR released the first edition of the Breast Imaging Reporting and Data System (BI-RADS), which standardized the terminology and assessment classification of mammography and built a unified communication framework for radiologists and clinicians [17]. After five updates, BI-RADS has become a global standard (Table 1). The implementation of the MQSA and the development of BI-RADS have greatly promoted the establishment of a standardized mammography QC system, realizing the transition from scattered technical operations to institutionalized quality management.

### 2.3. Development of Mammography QC in the Digital Era

The revolutionary digital mammography technology was approved by the U.S. Food and Drug Administration (FDA) in 2000 [19], which enabled post-processing of imaging data and the development of computer-aided detection and diagnosis (CAD) [20] and promoted the shift in QC from the performance assessment of screen-film systems to the refined control of the entire image acquisition–processing–display chain [21]. In 2003, Italian researchers developed the first phantom for periodic QC measurements of digital mammography systems to assess the reproducibility of parameters such as detector linearity, uniformity, image contrast, and spatial resolution [22]. The American College of Radiology Imaging Network (ACRIN) has conducted the Digital Mammography Imaging Screening Trial (DMIST) since 2001, and the FDA has implemented the “Enhancing Quality Using the Inspection Program (EQUIP)” since 2017 to ensure the optimal operation of digital equipment and the effectiveness of image quality [23,24]. Studies have demonstrated that structured audit logs within the EQUIP framework effectively enhance the performance of QC [24], but extra views at screening mammography increased with EQUIP implementation [25]. In March 2023, the FDA released a revised version of the MQSA (“2023 MQSA Final Rule”). A key update mandates that testing facilities provide patients with information about their breast density, thereby helping patients obtain important information that may affect their treatment decisions as completely as possible [26].

## 3. Factors Influencing Mammography Image Quality and Corresponding QC Measures

All steps of mammography are interconnected, ranging from patient management, breast positioning and compression, and image acquisition to image diagnosis and recommendations, record keeping, and result notification, as well as equipment performance testing, radiation dose optimization, and examination environment improvement [27]. Oversight in any link may affect the final image quality (Table 2).

### 3.1. X-Ray Equipment and Technical Parameters

The performance of mammography equipment and the accurate selection of technical parameters are important foundations for obtaining high-quality images. Appropriate X-ray tube targets and filtration materials have a significant impact on the X-ray spectrum, energy distribution, and image contrast. Combinations of wolfram (W) or molybdenum (Mo) targets with rhodium (Rh) or silver (Ag) filters are common clinical choices. Studies have shown that the W/Rh combination can provide better image quality and visibility of fibers and specks than the W/Ag combination, particularly at tube voltages of 30 and 32 kVp [28]. Meanwhile, breast thickness also affects the selection of target-filter combinations; for thin breasts, the Mo/Mo or Mo/Rh combinations yield better image quality [29]. The kVp and mAs are key parameters that determine the quality and quantity of X-rays. Under different breast thicknesses and tissue types, optimizing exposure parameters can reduce patient radiation dose while improving the image SNR [30]. The AEC system can automatically select the optimal exposure parameters to ensure the consistency of image density, but it is affected by multiple factors such as breast thickness, glandular composition, and body position [31]. In the era of digital mammography, image post-processing and display devices are also crucial links affecting image quality [21]. During a series of complex image processing processes (such as contrast enhancement, edge sharpening, and noise suppression), different algorithms can produce images with varying effects, which may enhance the visibility of lesions or introduce artifacts or distortions [33].

Regular performance evaluation and calibration of X-ray equipment are the focus of mammography QC, including the X-ray generation system (tube voltage, tube current, exposure time), detector performance (SNR, contrast-to-noise ratio, detective quantum efficiency), and image processing and display systems [50,51]. In early practices, image quality quantification was often achieved through manual periodic detection of the visibility of targets in standardized phantoms (such as the ACR-certified phantom, Contrast-Detail Mammography [CDMAM] phantom, and Polymethyl Methacrylate [PMMA] phantom) [34,35]. With technological advancements, the International Atomic Energy Agency (IAEA) proposed the integration of the ATAI to enable remote and automated quality control of mammography equipment in resource-limited regions, representing a new direction in performance monitoring [36]. It is necessary to test the repeatability of the AEC system and the consistency of exposure under different breast thicknesses to obtain appropriate image density under various clinical conditions [32]. In addition, ensuring that the brightness, contrast, and spatial resolution of diagnostic monitors comply with DICOM standards to provide an optimal image interpretation environment is also an important measure to improve the lesion detection rate [21].

### 3.2. Human Factors and Operational Procedures

**Figure 2:**
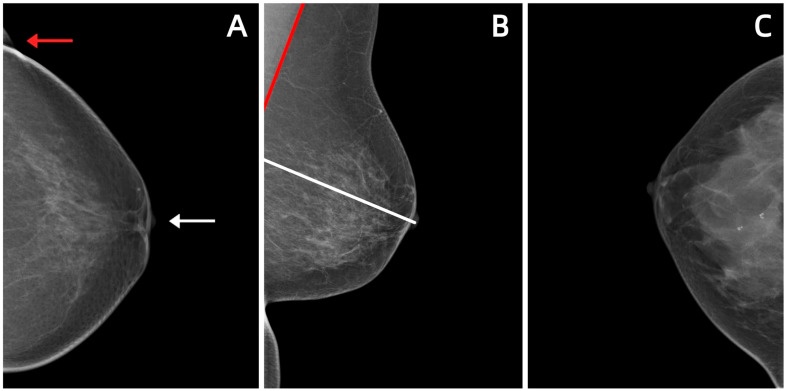
Common deficiencies in breast positioning during mammography: (**A**) Nipple not displayed in the tangential position (white arrow); presence of skin folds (red arrow). (**B**) The PNL (white line) did not reach the lower edge of the pectoralis major muscle (red line). (**C**) Inadequate visualization of the posterior adipose tissue behind the breast parenchyma.

**Figure 3:**
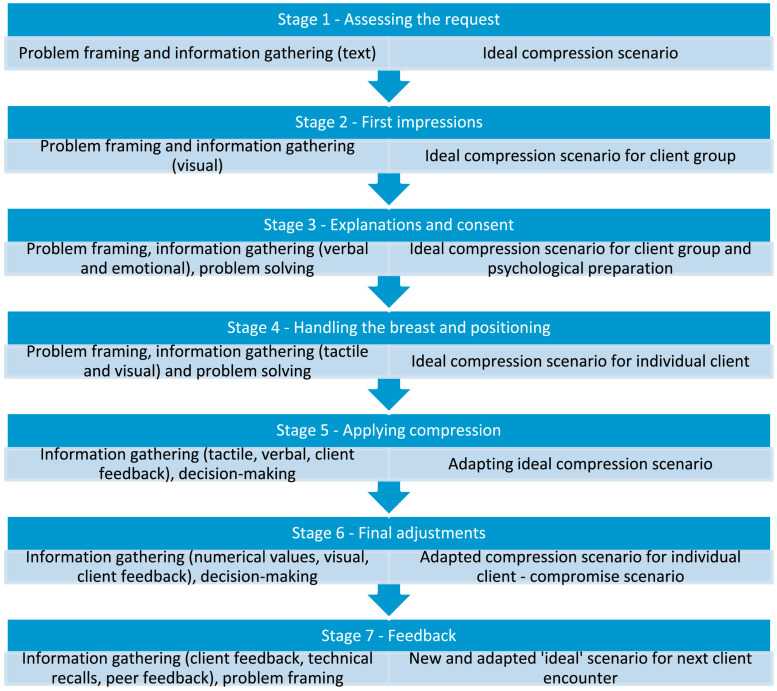
The seven-stage continuum mammography compression process model. The left-hand column indicates where different elements of problem analysis occur. The right-hand column identifies how the stages of problem-solving influence the ideal compression scenario. Reprinted with permission from: [53].

The technician’s professional skills, understanding of equipment performance, and mastery of patient positioning are direct factors in obtaining high-quality images [52]. Two key issues—patient positioning and breast compression—should be noted during mammography procedures, along with selecting appropriate imaging conditions, so as to obtain qualified images that meet diagnostic criteria (Table 3). Correct positioning is crucial for obtaining complete breast tissue images, especially ensuring the inclusion of the posterior part of the breast and the axillary extensions. The FDA has issued a statement pointing out that poor positioning is the main cause of most mammography image defects and clinical misdiagnoses [37]. Common deficiencies include non-tangential display of the nipple, insufficient visualization of the posterior breast tissue, the pectoralis major muscle not extending to the post-nipple line (PNL), and the presence of skin folds (Figure 2) [38,39]. Appropriate compression can not only reduce breast thickness and radiation dose but also homogenize tissue, reduce motion artifacts, and improve image contrast and sharpness [39]. Researchers have proposed a seven-stage breast compression problem-solving model that explains how the ideal compression scenario is identified and adjusted (Figure 3) [53].

To reduce the impact of human factors on image quality, regular training and testing should be conducted to ensure the standardization of operational procedures and the normalization of each examination. A survey involving 251 breast radiologists from 34 European countries showed that more than half of the respondents’ workplaces had established quality assurance measures, but less than one-third were required to participate in regular performance testing. Meanwhile, three-quarters of the respondents believed that mandatory testing would help improve their skills [42]. The fundamental purpose of establishing quality assurance measures lies in the implementation. Professional training on patient positioning techniques, selection of exposure parameters, and mastery of compression force, as well as timely updates to mammography operation guidelines, help improve the operational skills of technologists [43]. In addition, experienced radiologists can review the images acquired by technologists, identify and provide feedback on issues related to positioning, exposure, or post-processing through blind assessment, thereby promoting communication between radiologists and technologists [44].

### 3.3. Patient-Related Factors

The physiological characteristics and psychological state of patients also indirectly affect image quality. The shape, size, thickness, density, and composition (e.g., the ratio of adipose to glandular tissue) of the breast affect X-ray penetration and image contrast, thereby influencing image quality and diagnostic accuracy. Studies have found that when breast density changes from 6.6% to 33.5%, the probability of breast cancer detection decreases by 61%; when breast compression thickness changes from 46 mm to 66 mm, the probability of breast cancer detection decreases by 42% [45]. Discomfort and anxiety associated with mammography are important reasons why women avoid this examination. In particular, for women with implanted medical devices, not only is the contrast of breast images reduced and the projection of breast tissue and pectoralis major decreased, but patients also experience more pain and anxiety [49]. In addition, slight movement of patients during exposure can lead to image blurring, affect the clarity of microstructures (such as microcalcifications), and increase the risk of false negatives [46]. This is also the most common reason for technical recalls in screening mammography [47].

Improving patients’ compliance during the examination is conducive to improving image quality and promoting more extensive screening. The PAC device allows patients to adjust the compression force by themselves after initial compression by technologists. It can significantly improve the patient experience and achieve image quality similar to that of technologist-controlled compression [40]. It may even increase the additional compression force to reduce breast thickness and radiation dose [54]. Seventy-four percent of patients believe that the PAC device can encourage them to undergo re-examination [41]. Multi-sensory environmental upgrades to mammography rooms, such as playing soothing videos and diffusing light fragrances, are also helpful for improving patients’ psychological status, enabling higher PNL measurement values without increasing compression [48].

## 4. Limitations and Prospects of Mammography QC

### 4.1. Lack of Uniform Global QC Protocols

Currently, over 14 QC protocols for mammography are in use globally [55], and QC standards in regions such as the United States, Europe, Canada, Australia, and Japan have different focuses, with variations including the type of tests, phantoms used, image quality criteria, and limiting values. For instance, the digital mammography QC protocol drafted by the European Reference Organisation for Quality Assured Breast Screening and Diagnostic Services (EUREF) physicist team is a recognized European quality standard. Adopted by the European Federation of Organisations in Medical Physics (EFOMP), it is widely implemented in the Netherlands, the UK, Germany, and other countries. This protocol incorporates the CDMAM phantom into the QC testing system and establishes evaluation criteria for key dimensions, including contrast, spatial resolution, radiation dose, image quality, and acquisition repeatability [56]. However, France currently utilizes a protocol established by the French National Agency for Medicines and Health Products Safety (ANSM), which features more lenient QC thresholds and excludes the assessment of tomosynthesis and synthetic 2D (2DS) image quality [57]. This results in a heterogeneous situation and may affect the efficiency and accuracy of large-scale breast cancer screening [58].

In the future, mammography QC needs to pay more attention to international cooperation. By establishing unified examination standards, image metrics, and reporting specifications, the comparison and mutual recognition of examination results across different regions can be promoted. In resource-limited settings, a comprehensive evaluation of social benefits, economic costs, and health impacts should also be considered. This justifies the development of simplified quality control protocols to drive standardization and equity in global breast cancer screening [59].

### 4.2. Subjective Inconsistency in Image Quality Assessment

**Figure 4:**
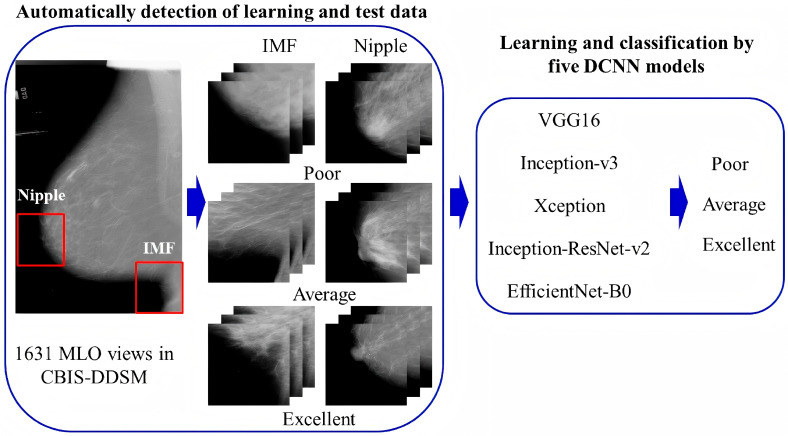
Working flow of constructing a DCNN model for breast positioning quality control and validation. It mainly includes automated detection of the learning and test data and learning and classification by five DCNN models. Reprinted with permission from: [69].

Mammography requires operators to have rich experience, and image quality assessment often relies on the subjective judgment of radiologists or technologists. Although various QC protocols provide basic standards for assessment, studies have shown that there is still subjectivity and inconsistency in quality assessment among different radiologists [60,61]. In recent years, artificial intelligence (AI) has advanced rapidly in breast imaging, with over 20 FDA-approved applications currently available [62]. However, these applications primarily focus on the diagnosis and differentiation of breast diseases [63,64], classification and typing [65,66], and evaluation of treatment efficacy [67,68], with limited use in QC. By analyzing massive image data through deep learning models, AI can automatically assess whether breast positioning is good, with accuracy rates of 96.5% and 93.3%, respectively, in the CC view and MLO view [37]. Other studies have also proposed a deep convolutional neural network (DCNN) classification for the quality control and validation of breast positioning criteria in mammography (Figure 4) [69].

Nevertheless, these studies are limited to single- or dual-center settings. The training of deep learning models is highly dependent on specific vendors, protocols, or regional datasets, leading to significant data dependency and poor generalization. Moreover, the lack of a unified evaluation framework—where some studies prioritize structural visibility while others focus on geometric accuracy—hinders direct performance comparisons across systems [70]. Therefore, future research should focus on developing a multi-dimensional, comprehensive AI model for evaluating mammography image quality. This model should integrate multiple assessment indicators, such as positioning accuracy, exposure parameters, image contrast, and microcalcification visualization. It is crucial to establish a standardized AI QC dataset for mammography, unify the evaluation criteria for image quality, and conduct multi-center clinical validation of the AI model to enhance its clinical applicability. Moreover, AI should be strictly positioned as an adjunct to radiologists rather than a substitute, preventing over-reliance on automation [57].

### 4.3. Imbalance Between Image Quality and Radiation Dose

While providing diagnostic information, mammography inevitably involves ionizing radiation exposure. Achieving a balance between the ALARA principle and high-quality images is an ongoing challenge [71]. Excessive pursuit of low doses may sacrifice image quality, leading to missed diagnoses; conversely, excessively high doses will increase the patient’s radiation risk. Existing QC procedures need to ensure that equipment maintains radiation dose at an acceptable level while providing optimal image quality [72].

Balancing diagnostic effects and radiation risks depends on the development of new materials and technologies. For example, compared with traditional broadband mammography, monochromatic X-ray sources are more sensitive for imaging breasts of various sizes and compositions and have lower radiation doses [73]. A composite shielding device composed of a 6 mm lead glass mask and a stainless steel compression plate can reduce radiation exposure to sensitive organs such as the lens and thyroid during mammography [74]. The advancement of new technologies reflects the “patient-centered” QC philosophy. Driven by the advancement of precision medicine, it is crucial to develop personalized dose optimization models based on patient-specific characteristics (such as breast density, thickness, and age), which enable dynamic adjustment of exposure parameters according to individual variations, thereby optimizing the trade-off between image quality and radiation dose [75].

### 4.4. Inapplicability of Traditional Standards to New Imaging Technologies

With the emergence of new technologies such as digital breast tomosynthesis (DBT) and contrast-enhanced mammography (CEM), traditional QC standards for two-dimensional mammography may no longer be fully applicable. For example, DBT reconstructs three-dimensional breasts through multi-angle X-ray images, which significantly reduces tissue overlap artifacts [76], but it also leads to prolonged imaging time and increased radiation dose. CEM obtains morphological information and blood supply of lesions through dual-energy projection, which can be used for diagnosis and lesion staging, but the use of contrast agents makes the QC process more complex [77].

New imaging technologies require specialized QC protocols. Currently, international QC protocols for DBT have been established by organizations such as EUREF, ACR, and the Australasian College of Physical Scientists and Engineers in Medicine (ACPSEM). The three protocols present a similar set of tests, focusing on collimation tests of light beams, X-ray tube and generator tests, AEC, dose information, image quality, and physical compression. Among them, the ACR standard is more suitable for adoption by other countries due to its high versatility, use of commercial phantoms, and independence from equipment manufacturers [78]. In parallel, countries such as Japan are actively researching and establishing performance assessment and QC procedures for DBT [79]. The French Society of Medical Physics (SFPM) has also established a working group to develop internal QC recommendations for DBT and 2DS images, preparing for the integration of DBT into breast cancer screening programs in the future [80]. Although an independent and comprehensive QC protocol specific to CEM has not yet been established, researchers are validating the utility of commercial and custom-made phantoms for CEM quality control [81,82,83]. Their results revealed that the PMMA phantom exhibited consistent quantification performance, whereas the commercial phantom was superior in visualization [83].

## 5. Conclusions

Over the past century, mammography and its QC have achieved remarkable development, which is essentially a process driven by the interaction between technological innovation and clinical needs. From the establishment of standards in the early stages to the precise and intelligent QC in the future, its core has always focused on improving image quality, reducing radiation dose, and enhancing patient experience. We have reason to believe that with the continuous development of AI and new imaging technologies, mammography—a traditional yet evolving technology—will benefit more patients.

## Figures and Tables

**Table 1:** BI-RADS assessment categories.

Assessment	Management	Likelihood of Cancer
Category 0: Incomplete—Need additional imaging evaluation and/or prior imaging for comparison	Recall for additional imaging and/or comparison with prior examination(s)	N/A
Category 1: Negative	Routine mammography screening	Essentially 0% likelihood of malignancy
Category 2: Benign	Routine mammography screening	Essentially 0% likelihood of malignancy
Category 3: Probably benign	Short-interval (6-month) follow-up or continued surveillance mammography	>0 but ≤2% likelihood of malignancy
Category 4: Suspicious	Tissue diagnosis	>2 but <95% likelihood of malignancy
Category 4A: Low suspicion for malignancy		>2 to ≤10% likelihood of malignancy
Category 4B: Moderate suspicion for malignancy		>10 to ≤50% likelihood of malignancy
Category 4C: High suspicion for malignancy		>50 to <95% likelihood of malignancy
Category 5: Highly suggestive of malignancy	Tissue diagnosis	≥95% likelihood of malignancy
Category 6: Known biopsy-proven malignancy	Clinical follow-up with surgeon and/or oncologist, and definitive local therapy (usually surgery) when clinically appropriate	N/A

N/A, not applicable. Reprinted with permission from: [18].

**Table 2:** Factors influencing mammographic image quality, core QC measures, and related studies.

Category of Influencing Factors	Specific Influencing Factors	Core QC Measures	Related Studies
X-ray equipment and technical parameters	Target-filter combination	Select the suitable combination according to breast thickness and calibrate the equipment regularly	Alkhalifah et al. [28]
			Kim et al. [29]
	Exposure parameters	Optimize parameters based on breast characteristics and follow the ALARA principle	Williams et al. [30]
	AEC system	Test system repeatability to ensure exposure consistency	Kattar et al. [31]
			Szczepura et al. [32]
	Image post-processing and display	Adopt standardized algorithms and ensure monitors comply with DICOM standards	Young et al. [21]
			Fausto et al. [33]
	Overall equipment performance	Regular detection with standardized phantoms and remote QC through ATAI	Figl et al. [34]
			Oberhofer [35]
			Mora et al. [36]
Human factors and operational procedures	Breast positioning	Implement standardized positioning for CC and MLO views	Brahim et al. [37]
			Feigin [38]
			Bassett et al. [39]
	Breast compression	Standardize compression operation and apply a PAC device if needed	Bassett et al. [39]
			Dontchos et al. [40]
			Balleyguier et al. [41]
	Professional competence of operators	Conduct regular training and assessment; radiologists conduct a blind review and provide feedback	Michalopoulou et al. [42]
			Tirada et al. [43]
			Sá Dos Reis et al. [44]
Patient-related factors	Breast physiological characteristics	Optimize imaging parameters based on breast characteristics and conduct patient education	Strandberg et al. [45]
	Patient psychology and movement	Optimize the examination environment and guide the patient to fix the body before exposure	Abdullah et al. [46]
			Martaindale et al. [47]
			Sarquis-Kolber et al. [48]
	Implantable medical devices	Optimize imaging position/parameters and reduce compression force selectively	Paap et al. [49]

ALARA, as low as reasonably achievable; AEC, automatic exposure control; DICOM, Digital Imaging and Communications in Medicine; ATAI, Automated Tool for Image Analysis; CC, craniocaudal; MLO, mediolateral oblique; PAC, patient-assisted compression.

**Table 3:** Photographic key points and image qualification criteria for CC view and MLO view.

		CC View	MLO View
Photographic key points	Positioning	The patient faces the mammography machine and turns the face to the non-examined side, with the examined arm hanging down and externally rotated. The breast is placed at the center of the imaging plate with the nipple in a tangential position, and equal spacing is maintained on the medial and lateral sides of the breast.	The patient faces the mammography machine with feet naturally apart. The imaging plate is angled at 30–60° to the horizontal plane, compressing and fixing the examined breast and the ipsilateral anterior axillary fold (including the upper-outer portion of the pectoralis major muscle). The imaging plate is parallel to the pectoralis major muscle, reaching the upper edge of the patient’s axilla. The outer-upper corner vertex of the imaging plate is directly opposite the apex of the examined side’s axilla.
	Imaging range	Includes bilateral (or unilateral) full breast skin from medial to lateral aspects.	Includes the soft tissue under the examined side’s axilla and the skin below the breast
	Central ray	X-rays are projected from cranial to caudal.	X-rays are projected from the inner-upper to the outer-lower direction.
	Exposure conditions	25–35 kVp, with automatic exposure control or automatic parameter selection.	
Image qualification criteria		The base of the breast should be included, with as much of the anterior edge of the pectoral muscle displayed as possible.	The pectoralis major muscle should be fully displayed, with its lower edge extending to or below the post-nipple line.
		The difference in the length of the post-nipple line between CC and MLO views should be ≤1 cm.	The inframammary fold should be unfolded and distinguishable.
		The CC images of bilateral breasts should appear relatively spherical.	The left and right breast images should be placed back-to-back symmetrically in a diamond shape.
		The adipose tissue behind the breast parenchyma should be fully displayed.	
		The nipple should be in a tangential position without overlapping with fibroadenomatous tissue.	
		No skin folds should be present.	
		The image should have distinct layers, with clear lesion display, capable of showing fine calcifications of 0.1 mm.	

## Data Availability

No new data were created or analyzed in this study. Data sharing is not applicable to this article.
